# Sonodelivery Facilitates Sustained Luciferase Expression from an Episomal Vector in Skeletal Muscle

**DOI:** 10.3390/ma8074608

**Published:** 2015-07-22

**Authors:** Manoel Figueiredo Neto, Rachel Letteri, Delphine Chan-Seng, Todd Emrick, Marxa L. Figueiredo

**Affiliations:** 1 Department of Pharmacology & Toxicology, 301 University Blvd., Galveston, TX 77555, USA; E-Mail: mafiguei@utmb.edu; 2 Department of Polymer Science and Engineering, University of Massachusetts-Amherst, Amherst, MA 01003, USA; E-Mails: rletteri@alumni.nd.edu (R.L.); tsemrick@mail.pse.umass.edu (T.E.); 3 Institut Charles Sadron UPR22-CNRS, 23 Rue du Loess, 67034 Strasbourg, France; E-Mail: Delphine.Chan-Seng@ics-cnrs.unistra.fr

**Keywords:** sonodelivery, episome, polymer, nanoplex, microbubbles, non-viral vector, skeletal muscle, sustained expression, luciferase

## Abstract

Successful gene delivery to skeletal muscle is a desirable goal, not only for treating muscle diseases, but also for immunization, treatment of metabolic disorders, and/or delivering gene expression that can treat systemic conditions, such as bone metastatic cancer, for example. Although naked DNA uptake into skeletal muscle is possible, it is largely inefficient in the absence of additional chemical or physical delivery methods. We describe a system for delivery of non-viral or plasmid DNA to skeletal muscle using ultrasound-assisted sonoporation of a nanoplex combining plasmid DNA and a branched polymer based on poly(cyclooctene-*graft*-oligopeptide). The materials and methods described herein promise to advance the field of sonodelivery and of gene delivery to muscle for therapeutic applications since a simple system is presented that enables long-term gene expression *in vivo* with the promise of a minimal inflammatory gene expression profile.

## 1. Introduction

Successful gene delivery to skeletal muscle is a desirable goal, not only for treating muscle diseases, but also for immunization, and/or delivering gene expression for treating systemic conditions such as bone metastatic cancer or metabolic disorders. Although naked DNA uptake into skeletal muscle is possible, it is largely inefficient in the absence of additional chemical or physical delivery methods. We have recently described a system for delivering plasmid DNA to skeletal muscle using ultrasound-assisted sonoporation (sonodelivery) of a nanoplex which combines plasmid DNA and a branched polymer, poly(cyclooctene-graft-oligopeptide) [1,2]. 

In the present report, our main objective was to examine the potential for long-term expression of an episomal vector in skeletal muscle following sonodelivery. We utilized a plasmid that is capable of long-term maintenance within cells as an episomal vector. Episomal vectors are extra chromosomal eukaryotic vectors that represent a promising alternative to traditional viral gene therapy vectors, which typically require integration into the host genome to provide long-term gene expression. Episomes avoid insertional mutations that may be caused by integration events into the host genome. The precursor to the vector utilized in our study was first described as pEPI-1, a nonviral and episomally-replicating vector [3]. pEPI-1 was maintained episomally due to an active transcription unit which was linked to a scaffold/matrix attachment region, also known as S/MAR or MARS. MARS regions take part in forming chromatin domains and function as an origin of replication support. MARS can aid in the efficient retention of episomes and also transgene expression in mammalian cells. pEPI replicates autonomously in various cell lines, even in the absence of selective pressure. However, it has a limited use in gene therapy due to its low efficiency establishing itself as a replicating episomal entity soon after delivery into the targeted cells—nearly all plasmids are lost during ensuing cell division, and therefore few of the originally transfected vector molecules are capable of replicating episomally in a stable manner. One successful example of a modified pEPI is the vector pEPI-eGFP. Studies have shown that changes in chromatin state of the host, as well as of the vector, cause changes in episomal gene activity and influences the episome’s distributions into nuclear compartments. Therefore, episomal genes are subject to control systems of the host, as is shown for their equivalents in the host genome. The vector we utilized in the present report has been shown to be a very effective version of pEPI. This is the pEPIto, a vector with several modifiable regions, providing flexibility in experimental design and therapeutic gene expression profile. The prolonged transgene expression profiles of pEPito-based vectors *in vivo* appears to result from a combination of reduced epigenetic silencing due to the modified bacterial vector backbone, and the MARS region, which might either trigger the translocation of vector molecules to sites of active chromatin, or enhance overall transcription levels [4]. 

Episomal vectors based on pEPI are continuously being refined and we envision that episomally replicating vectors will have applications for replicating in a tissue specific manner. For example, the human AFP-promoter, combined with the hCMV enhancer element, has demonstrated to be a valid tissue-specific promoter targeting certain carcinomas, and tissue-specific replication was demonstrated *in vitro* with the muscle-specific SM22 promoter. Combining a tissue specific pEPIto vector system with appropriate delivery systems will lead to higher tissue-specificity, diminishing undesired consequences and proving to be suitable for long term transgene expression *in vivo* within gene therapy.

## 2. Results and Discussion

### 2.1. An Episomal Plasmid Can Be Complexed with a Polymer and Delivery Is Enhanced by Ultrasound in Skeletal Muscle Cells

**Figure 1:**
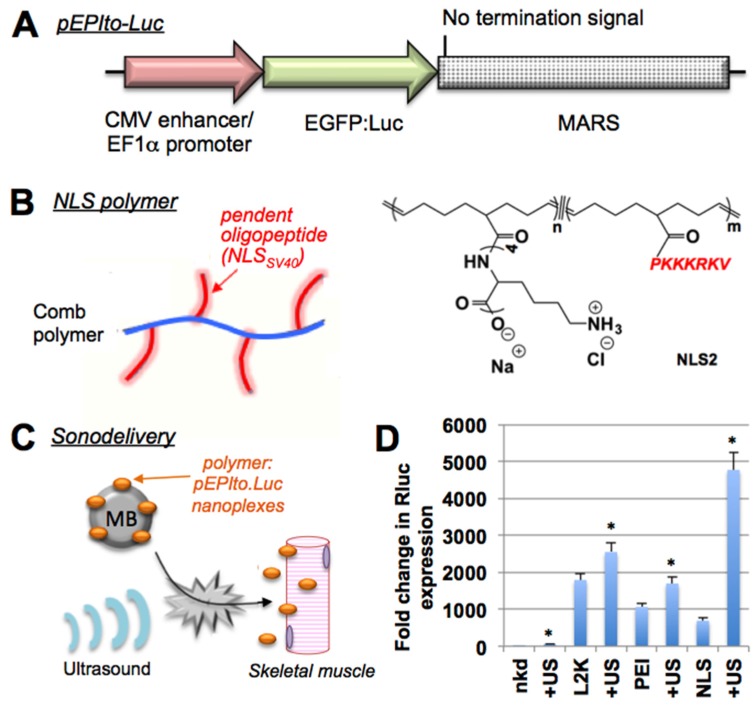
(**A**) pEPIto-Luc, containing a cytomegalovirus (CMV) enhancer and an elongation factor 1 alpha (EF1a) promoter driving expression of an enhanced green fluorescent protein andfirefly luciferase fusion gene (EGFP:Luc). The plasmid is maintained as an episome due to the presence of a scaffold/matrix attachment region (MARS) which follows the Luc coding region in the absence of a termination signal. (**B**) The NLS2 polymer used for gene delivery *in vivo*. The polymer is composed of a poly(cyclooctene-*graft*-oligopeptide) comb or branched structure which enhances DNA complexation and release once inside cells. (**C**) The sonodelivery system. A nanoplex is delivered to skeletal muscle via complexation of the NLS2 polymer with the pEPIto-Luc plasmid, then delivered through microbubble-assisted sonoporation via ultrasound stimulation. The nanoplexes enter the muscle and are transported to the nucleus through the NLS moiety in the polymer. (**D**) Transfection of C2C12 skeletal muscle cells is significantly enhanced by ultrasound (+US) compared to each -US control for naked, lipofectamine 2000 (L2K) and NLS2 reagents, but not PEI (*, *p* < 0.05).

An episomal plasmid was used for gene delivery that contains the CMV enhancer and an elongation factor 1 alpha promoter (CMV/EF1a), as well as a reporter gene fusion of enhanced green fluorescent protein and firefly luciferase (EGFP:Luc), followed by a scaffold/matrix attachment region (MARS) (Figure 1A). Surprisingly to us and others, the MARS region appears to play a role in long term gene expression in muscle. Another group has reported long-term expression from an earlier version of the episome vector (pEPI1-luc) in muscle. They showed data for persistent luciferase expression for ~84 days from the episome and no expression for a traditional vector [5]. It is possible that the advantage of the MARS element in muscle is not the replication ability per se, as it is a post-mitotic structure, but rather that the pEPI is less toxic and/or modulates less immune modulation, enabling preservation in muscle fibers for a significantly longer time than a traditional plasmid. 

We utilized a polymer with a comb architecture, which efficiently complexes plasmid DNA for gene delivery. This type of polymer, has PKKKRKV heptapeptide nuclear localization signal (NLS) sequences to provide a branched poly(cyclooctene-graft-oligopeptide) or NLS2, which enhances gene delivery in the presence of ultrasound [2] (Figure 1B). We have found that ultrasound stimuli can help enhance gene delivery of this nanoplex (pDNA:NLS2) to muscle cells in the presence of microbubbles that induce cavitation (Figure 1C). In this study, we have examined the efficiency of transfection of the NLS2 in skeletal muscle cells, C2C12 (Figure 1D), and observed that although ultrasound (US) greatly augments the efficiency of commercial transfection reagents lipofectamine 2000 (L2K) or naked DNA, the effect of US on NLS2 transfection efficiency is enhanced by a further two-fold compared to L2K (*, *p* < 0.05 compared to no ultrasound controls for each group). These results suggested that ultrasound, combined with the NLS2 polyplex can be a very useful method of gene delivery for plasmid DNA in muscle cells.

### 2.2. Sonodelivery of pEPIto-Luc in Vivo Yields Long-Term Gene Expression in Skeletal Muscle

**Figure 2:**
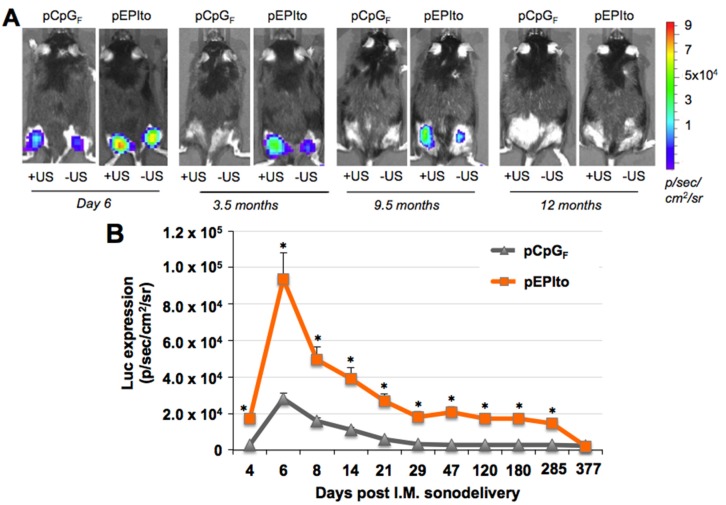
(**A**) Sonodelivery of pEPIto-Luc *in vivo*; (**B**) Quantification of pEPIto-Luc sonodelivery *in vivo* over time. Signals detected using IVIS Xenogen and presented in p/sec/cm^2^/sr. *, *p* < 0.05 relative to pCpG_F_ control values at each time point measured.

We next examined ultrasound-assisted or sonodelivery of the NLS2:pDNA nanoplexes *in vivo* by using either pCpG_F_ or pEPIto vectors expressing the reporter gene luciferase. Ultrasound stimuli (+US) enhanced the transfection efficiency to hind thigh skeletal muscle for both vectors by day 6 (Figure 2A), although pEPIto was able to enable luciferase expression at higher absolute levels compared to pCpG_F_ (p/sec/cm^2^/sr; *, *p* < 0.05) (Figure 2B). We also were able to examine expression over time, from day 6 to past 12 months of age. While pEPIto supported expression of luciferase up do day 285 (~9.5 months), the pCpG_F_ vector only supported detectable reporter gene expression up to ~day 21 (Figure 2B). These results indicated that pEPIto can support long-term expression of a reporter gene, which should enable sustained gene therapy efforts for controlling systemic conditions such as metabolic defects or bone-metastatic cancers.

### 2.3. pEPIto Does Not Promote an Inflammatory Response in Muscle Following Sonodelivery

**Figure 3:**
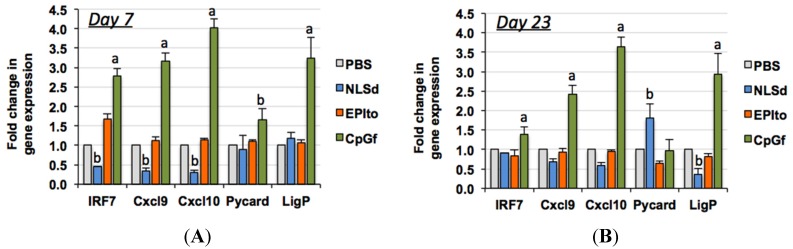
*In vivo* delivery of pEPIto in skeletal muscle promotes gene expression changes but these are consistent with a reduced inflammatory response by day 7 and day 23 post-delivery as compared to pCpG_F_ control. (**A**) *p* < 0.05 relative to all other groups; (**B**) *p* < 0.05 relative to phosphate-buffered saline control (PBS) (ANOVA).

As assessed by gene expression and histology analyses, pEPIto administration does not appear to promote an inflammatory response in skeletal muscle *in vivo*. These observations compared pEPIto with phosphate-buffered saline control (PBS), polymer (NLS), or pCpG_F_. We selected genes that encode signaling molecules previously linked with potential inflammatory responses to plasmid DNA [6], including interferon (IFN) response factor (IRF) transcription factors that orchestrate the innate immune response as well as other genes related to the upregulation of other immune system elements such as chemokines. Quantitative real-time PCR (qPCR) showed that compared to PBS control, the NLS2 polymer mostly down regulated the expression of genes associated with an inflammatory response in skeletal muscle. For example, polymer alone down regulated genes associated with innate immune signaling (IRF7), IFN response (LigP), and chemokines (CXCL9 and 10) (Figure 3A). However, polymer upregulated Asc/PYCARD, a gene of the innate immune system responsible for assembly of large signaling complexes in inflammatory pathways. Surprisingly, pEPIto had a more favorable profile concerning inflammatory gene expression signature than a traditional, pCpG-free (pCpG_F_) vector (Figure 3A). pCpG_F_ upregulated all inflammation-related genes examined with the exception of PYCARD. pEPIto, on the other hand, down regulated CXCL9, PYCARD, and LigP genes, while the expression of IRF7 and CXCL10 remained unchanged relative to PBS control. These results would suggest that the pEPIto might be preferable to pCpG free vectors, which appear to induce a variety of pathways related to an inflammatory response.

**Figure 4:**
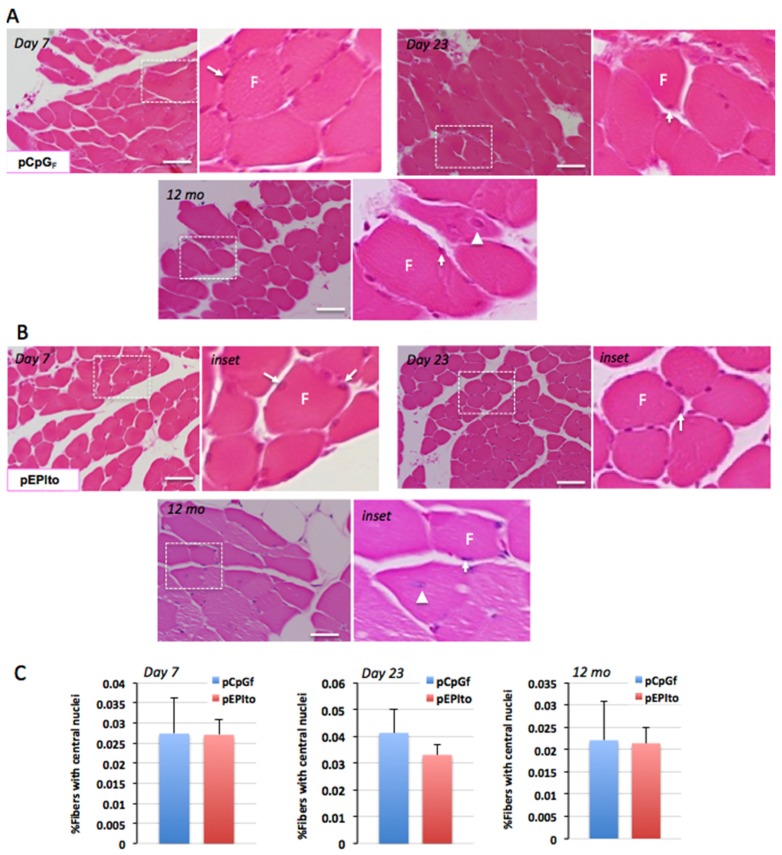
Histological analyses at day 7, 23, and 12 months indicate neither pCpG_F_ (**A**) nor pEPIto (**B**) produce any significant signs of damage, as shown by H&E staining and visualization of skeletal muscle at 200x magnification. Inset, selected areas at higher magnification to show nuclear positioning in muscle fibers. Scale bar, 50 μm. Arrows, normal positioning of nuclei at the periphery of muscle fibers (F); arrowheads, occasional atypical nuclei positioned centrally within fibers. (**C**) Analyses of percentage of muscle fibers displaying atypical central nuclei show no significant differences (*p* > 0.3) for either plasmid group for all three time points.

The morphology of skeletal muscle was assessed at day 7, 23, and at endpoint, *i.e.*, ~12 months post-sonodelivery (Figure 4A,B). Analyses of percentage of H&E stained muscle fibers displaying atypical central nuclei show no significant differences (*p* > 0.3) for either plasmid group for all time points (Figure 4C). Neither pCpG_F_ nor pEPIto produced any local or systemic signs of prior or existing damage. This data would suggest that this vector and mode of delivery might be relatively safe and potentially translatable to clinical studies that require long-term gene expression. Additionally, the pEPIto vector may be a viable alternative to viral vectors, which have the disadvantages of requiring integration into the genome for sustained gene expression, and may promote inflammation within muscle [7].

## 3. Experimental Section

### 3.1. Vectors

Plasmid DNA vectors included pEPIto (PlasmidFactory, Bielefeld, Germany) or pCpGFree-mcs (pCpG_F_) (Invivogen, San Diego, CA, USA) backbones expressing firefly luciferase and green fluorescent protein genes (Luc, GFP). The Luc-GFP cassette was cloned by PCR cloning into pCpG_**F**_ using NheI and BglII ends in order to produce the same expression cassette (CMV enhancer and EF1 promoter) in pCpG_**F**_. Vectors were prepared for all experiments using Endofree kits (Qiagen, Valencia, CA, USA). For efficient complexation with polymer, vectors were first precipitated and resuspended in water. Briefly, precipitation used 1:10 volume 3M NaOAc and 2 volumes of cold 100% Ethanol, followed by a 30 min incubation at −80 °C and centrifugation at 12,000 rpm for 15 min at 4 °C, and a wash using 2 volumes of 70% Ethanol with a 5 min spin at room temp. The pellet was allowed to dry and was resuspended in sterile nuclease free water.

### 3.2. In Vivo Studies

Animal care and procedures were performed in accordance with the UTMB institutional review board guidelines. For gene delivery, we used a polymer containing the Simian Virus SV40 nuclear localization signal (NLS) NLS2, a comb shaped polymer with a polycyclooctene backbone and tetralysine and NLS oligopeptide pendent groups (~50 mol% of each oligopeptide). The synthesis of NLS2 is described in [1]. We prepared polymers in low retention Eppendorf tubes, dissolved in nuclease-free water, and sterilized by filtration. The stock solution of NLS2 was diluted to enable complexation with pLuc plasmid DNA at an *N*/*P* ratio = 6. The ratio of protonatable nitrogens in the polymer, *N*, to DNA phosphates, *P*). The protonatable nitrogens are the amines in the polymer pendent chains, distinct from the amide nitrogens of the polypeptide chain that are not protonatable under these conditions. DNA (12.5 μg) was added to nuclease-free water 1:1 to polymer solution and allowed to equilibrate for a minimum of 35 min under sterile conditions. Following polyplex formation, 5.5% sterile Micromarker microbubbles (VisualSonics, Toronto, ON, Canada) were added per tube and injected intramuscularly in 50 μL to the hind legs of anesthetized C57/BL6 8-week-old male mice (5 per group). After applying ultrasound gel, we irradiated the muscle to mediate sonoporation and thus gene delivery of Luc plasmids using a Sonigene sonoporator (VisualSonics) using settings of 1 MHz, 20% duty cycle, 3 W/cm^2^, 60 s. *In vivo* imaging for luciferase expression in muscle was performed starting on day 6 following sonoporation using previously published procedures by intravenous luciferin substrate administration and collection of images within 10–20 min [8,9].

### 3.3. Real-Time Quantitative RT-PCR (qPCR) Analyses

Total RNA from 10–30 mg muscle isolated from the mouse hind thigh was extracted using polytron homogenization in Trizol as described [10], followed by purification using a SurePrep kit (FisherSci). One microgram of RNA was reverse-transcribed using *amfiRivert* Platinum cDNA Synthesis Master Mix (GenDEPOT, Barker, TX, USA). Real-time qPCR reactions contained 1 µL cDNA template, 2× SYBR Green Master Mix (Applied Biosystems, Foster City, CA, USA), and 10 µM forward and reverse primers for both experimental and β-actin controls. qRT-PCR was performed on an Eppendorf Realplex 2S (Eppendorf, Hauppauge, NY, USA), using: 40× 95 °C for 3 min; 95 °C for 3 s; 60 °C 30 s; 72 °C 8 s and analyzed using EP Realplex software (Ver. 2.2). 

### 3.4. Histology

Hind thigh muscles were fixed overnight in 10% buffered formalin (Fishersci), then embedded in paraffin and sectioned (5 μm). Sections were stained with hematoxylin and eosin and imaging was performed using a photomicroscope at the Research Histopathology Core. 

### 3.5. Statistical Analysis

Quadruplicate wells were used in real time PCR analyses and experiment repeated twice, with values provided as mean ± SEM or 95% confidence interval. Comparisons for transfection data were performed using an unpaired t-test, and *p* < 0.05 was considered to indicate a significant difference. Comparisons for the real-time PCR gene expression data used one-way ANOVA analyses with α = 0.05, with a Tukey’s test for pairwise comparison of group means.

## 4. Conclusions 

Most recently, a tissue-specific episomal version of pEPI has been described [11] that has high promise for extending the applications of present vectors towards more efficient gene therapy and gene delivery applications. The delivery system and vector described herein promise to advance the field of sonodelivery and of muscle gene delivery for therapeutic applications. A simple approach is shown which can achieve long-term gene expression *in vivo* with the promise of a minimal profile of inflammatory gene expression. Episomal vectors based on pEPI have continued to be refined and we envision that episomal vectors will have applications for replicating in a tissue specific manner. Combining a tissue specific pEPIto vector system with appropriate delivery systems will lead to higher tissue-specificity, diminishing undesired consequences and proving to be suitable for long term transgene expression *in vivo* within gene therapy.
